# Are Artificial Intelligence recommendations in medical practice reliable?

**DOI:** 10.31744/einstein_journal/2026CE2451

**Published:** 2026-10-13

**Authors:** Thalya Fernanda Horsth Maltarollo, Lucas Domingues-dos-Santos, Caleb Shitsuka, Mary Caroline Skelton-Macedo

**Affiliations:** 1 Universidade de São Paulo Faculdade de Odontologia Department of Dentistry São Paulo SP Brazil Department of Dentistry, Faculdade de Odontologia, Universidade de São Paulo, São Paulo, SP, Brazil.; 2 Universidade Federal do Espírito Santo Faculdade de Odontologia Department of Clinical Dentistry Vitória ES Brazil Department of Clinical Dentistry, Faculdade de Odontologia, Universidade Federal do Espírito Santo, Vitória, ES, Brazil.

Dear Editor,

Medical practice is accompanied by a number of challenges and must be based on scientific evidence in order to make safer and more efficient decisions. In this scenario, Artificial Intelligence (AI) has the potential to play an essential role in assisting healthcare professionals who need to choose between different treatment options. It summarizes and interprets scientific literature, influencing decision-making and improving personalized patient care.^(1)^

 Currently, AI is advancing rapidly. It encompasses a range of technologies that enable machines to perform advanced functions, including learning, reasoning, understanding, analyzing data, and providing recommendations, abilities that were previously exclusive to humans. Although this technology offers unprecedented speed in acquiring information, its use in practice requires careful analysis of its accuracy and reliability.^(2)^

One of the main problems with AI is something called "hallucinations", in which the system generates information that appears plausible but is inaccurate or fabricated. This occurs because it is designed to prioritize the generation of coherent and seemingly accurate responses, often favoring probabilistic "guesses" rather than explicitly recognizing uncertainty.^(3)^ This behavior can be observed when models like ChatGPT invent references or incorrectly interpret established clinical guidelines. However, clinical practice depends on highly specific and rigorously validated evidence, and the uncritical use of AI-generated results without verification protocols poses a risk to patient health.^(4)^

To minimize these risks, healthcare professionals should go beyond superficial interactions with AI and adopt prompt engineering. This approach refers to the strategic formulation of inputs to guide the model's reasoning process and serves as a filter against erroneous outputs.^(5)^ In medical contexts, the use of improvised and unstructured AI is generally inappropriate. Instead, the adoption of organized structures and Retrieval-Augmented Generation (RAG) systems is necessary. RAG architectures are particularly relevant to evidence-based practice, as they restrict AI responses to information retrieved from verifiable databases, such as Scopus and Web of Science, helping reduce hallucination and error rates compared with standard generative models.^(6)^

It is well known that AI has considerable potential to assist clinical practice. However, these benefits are more appropriately achieved through specific models or specialized systems rather than generic AI tools. Because the precise formulation of instructions is critical to error mitigation, these systems may allow healthcare professionals to focus on clinical reasoning while ensuring access to reliable and validated evidence. Ultimately, AI should be used as a support tool, not as a substitute for scientific knowledge or critical thinking. The responsibility for critical evaluation and clinical decision-making remains unequivocally with the dental professional.

## Data Availability

The content is already available.
